# Nucleosome architecture throughout the cell cycle

**DOI:** 10.1038/srep19729

**Published:** 2016-01-28

**Authors:** Özgen Deniz, Oscar Flores, Martí Aldea, Montserrat Soler-López, Modesto Orozco

**Affiliations:** 1 Institute for Research in Biomedicine (IRB Barcelona). Baldiri Reixac 10-12. 08028 Barcelona, Spain; 2 Joint BSC-CRG-IRB Program in Computational Biology. Baldiri Reixac 10-12. 08028 Barcelona, Spain; 3 Molecular Biology Institute of Barcelona (IBMB) CSIC. Baldiri Reixac 4. 08028 Barcelona, Spain; 4 Department of Biochemistry and Molecular Biology. University of Barcelona, 08028 Barcelona, Spain

## Abstract

Nucleosomes provide additional regulatory mechanisms to transcription and DNA replication by mediating the access of proteins to DNA. During the cell cycle chromatin undergoes several conformational changes, however the functional significance of these changes to cellular processes are largely unexplored. Here, we present the first comprehensive genome-wide study of nucleosome plasticity at single base-pair resolution along the cell cycle in *Saccharomyces cerevisiae*. We determined nucleosome organization with a specific focus on two regulatory regions: transcription start sites (TSSs) and replication origins (ORIs). During the cell cycle, nucleosomes around TSSs display rearrangements in a cyclic manner. In contrast to gap (G1 and G2) phases, nucleosomes have a fuzzier organization during S and M phases, Moreover, the choreography of nucleosome rearrangements correlate with changes in gene expression during the cell cycle, indicating a strong association between nucleosomes and cell cycle-dependent gene functionality. On the other hand, nucleosomes are more dynamic around ORIs along the cell cycle, albeit with tighter regulation in early firing origins, implying the functional role of nucleosomes on replication origins. Our study provides a dynamic picture of nucleosome organization throughout the cell cycle and highlights the subsequent impact on transcription and replication activity.

The nucleosome, a nucleo-protein complex composed of 147 bp of double stranded DNA wrapped around a histone octamer, is the fundamental structural unit of chromatin in eukaryotic organisms^1^,^2^. Besides packaging DNA, nucleosomes play a major role in controlling DNA accessibility to regulatory proteins, hence affecting cellular processes such as transcription, DNA replication and repair^3^,^4^,^5^.

Genome-wide studies^4^,^6^,^7^,^8^,^9^,^10^,^11^,^12^ have shown that nucleosomes are not randomly positioned along the genome, but they are highly abundant at some positions, while rare in others. In the best-studied organism, budding yeast, nucleosomes show a canonical pattern around transcription start sites (TSSs) and replication origins (ORIs)^8^,^9^,^13^,^14^,^15^,^16^, where a well-defined nucleosome free-region (NFR) is surrounded by two well-positioned nucleosomes, defining the phase of the nucleosome string^17^.

Earlier studies have suggested that the presence of nucleosomes in the promoter region is related to gene inactivation^1^,^8^,^18^,^19^,^20^,^21^,^22^,^23^. In line with that, the replacement of sequences at TSS-NFR by strong nucleosome-favoring sequences has been shown to inhibit transcription^24^. However, deep analyses of nucleosome organization around TSSs in different organisms have revealed that the connection between nucleosome architecture and DNA transcription is much more complex than originally anticipated, and that high nucleosome density around TSSs does not necessarily interfere transcription^17^,^25^,^26^,^27^,^28^,^29^. Moreover, various studies have demonstrated similar complexity in the interplay between replication activity and nucleosome architecture around ORIs.^9^,^16^,^30^,^31^,^32^,^33^,^34^.

Furthermore, for faithful DNA replication and transcription nucleosome organization should display well-regulated rearrangements along the cell cycle at ORIs and TSSs, as chromatin structure undergoes global conformational changes^35^,^36^,^37^,^38^,^39^,^40^,^41^,^42^. However, the nature of these changes, and the relationship between nucleosome plasticity and DNA functionality throughout cell cycle is still unclear^43^,^44^.

We present here to the best of our knowledge the first comprehensive genome-wide study of nucleosome plasticity along the *S. cerevisiae* cell cycle at single base-pair resolution. We focused our analysis on cell cycle plasticity of nucleosome architecture around key regions: transcription start/termination sites and replication origins. Our study provides novel insights into the connection between nucleosome rearrangements and cell cycle-related dynamics in DNA replication and transcription.

## Results and Discussion

### General nucleosome architecture around TSSs and ORIs

**Figure 1:**
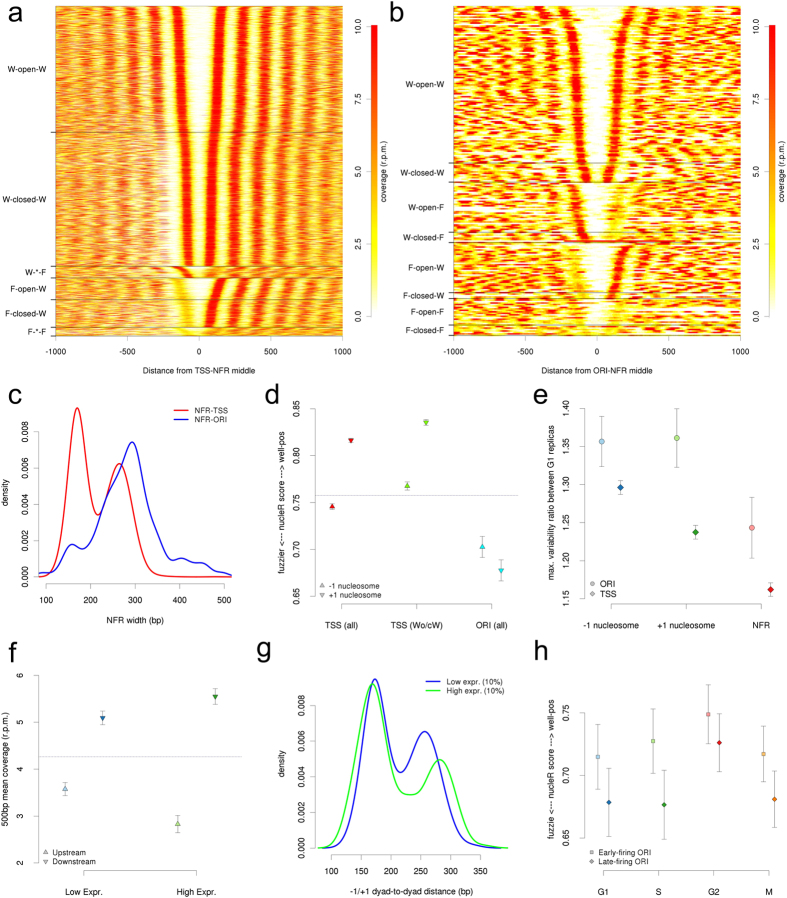
Nucleosome organization around TSSs and ORIs at G1 phase. (**a**) Heatmap of MNase-seq coverage (**a**) around TSSs (**b**) around ORIs (aligned taking strand directionality into account). Darker colors represent higher nucleosome occupancy. Rows are sorted according NFR-width and grouped by adjacent nucleosome classification (see methods for details). (**c**) Distribution of −1/ + 1 nucleosome dyads distance at TSSs and ORIs. While TSS-NFRs show a bi-modal distribution (peaks at 170 bp and 270 bp), ORI-NFRs are in general wider with a dyad-to-dyad distance of 300 bp (**d**) Mean nucleosome positioning scores (nucleR scores) of + 1/−1 nucleosomes at TSSs and ORIs. Value 1 indicates the highest coverage and phasing of nucleosomes, while value 0 points to disorganized and lowest-covered structure. Error bars, when visible, indicate the 95% confidence interval (CI); dashed horizontal line represents the global mean. (**e**) Mean fuzziness score and NFR width ratio between maximum and minimum values observed in triplicates. Higher values indicate more variability among G1 replicas. Error bars indicate the 95% CI (**f**) Comparison of the mean nucleosome coverage between the genes with constitutively low-expression and constitutively high-expression. Error bars indicate the 95% CI. (**g**) NFR distribution of the genes with constitutively low-expression and constitutively high-expression. (**h**) nucleR aggregated score for −1/ + 1 nucleosomes of early- and late-firing replication origins at each the cell cycle stage. Symbol indicates the mean nucleR score and error bars the 95% CI.

Our MNase-Seq datasets provide a 50× coverage across the yeast genome, increasing up to 70× across gene bodies and 86× when only the replication origins are considered. We have been able to map the average location of about 67000 nucleosomes, which occupy nearly 80% of the yeast genome. Nucleosome architectures around TSSs and ORIs have been classified based on the width of the NFRs (closed (c) or open (o)) and the positioning of the −1 and +1 nucleosomes (missed (M), fuzzy (F) or well-positioned (W)), as described earlier^17^. Overall, we could classify 81% of TSSs and more than 90% of ORIs nucleosome strings. Figure 1a,b demonstrate the average nucleosome configurations of three biological replicas collected at G1 phase, which are used for further comparison of nucleosome organization around TSSs and ORIs.

TSS-NFR width shows a bimodal distribution^17^,^39^ which corresponds to two nearly equally populated states: *closed* (a dyad-to-dyad distance between −1/ + 1 nucleosomes around 170 bp) and *open* (around 270 bp) architectures (Fig. 1a,c). On the contrary, ORI-NFRs are in general wider and the majority is annotated as “open” state (Fig. 1b,c). Like TSSs, previously demonstrated by our group^17^, ORIs wider NFRs are marked by high deformation energy, implying that the physical properties of these DNA sequences make them refractory to nucleosome formation (Sup. Figure S2a). This finding hence suggests that intrinsic properties of DNA are important to define not only TSS-NFRs^14^,^17^, but also ORI-NFRs. Of note, DNase I footprint data derived from the origin recognition complex (ORC) binding at replicating consensus sites (ACSs) reveal much shorter sites than ORI-NFRs^45^, demonstrating that ORC binding alone cannot define the boundaries of ORI-NFRs (Sup. Figure S2a). Moreover, the −1 and +1 nucleosomes are in general better positioned around TSSs than around ORIs based on nucleR scores (Fig. 1d), proposing a rather loose chromatin structure-based regulation of DNA replication. Interestingly, while nucleosome positioning around TSSs is asymmetric (i.e.+1 nucleosomes are better phased than −1 nucleosomes), nucleosomes around ORIs are symmetrically positioned (Sup. Figure S2b). A potential explanation would be that nucleosome symmetry around ORIs enables bidirectional DNA replication from the origins, while asymmetrical nucleosome organization around TSSs may facilitate unidirectional transcription.

### Inherent noise and fuzziness in nucleosome maps

As described in previous work^17^,^46^ nucleosome maps are intrinsically noisy, suggesting that nucleosomes are continuously sliding along the DNA fiber. Some nucleosomes are specially fragile, as such they may be captured in the nucleosome maps depending on the nucleosome preparation techniques, i.e. cross linking efficiency, MNase activity^47^,^48^,^49^. In order to estimate such “noise”, we have analyzed genome-wide nucleosome maps of G1 synchronized yeast population in three biological replicas. Provided that biosynthetic activities are at high rate in G1 phase, nucleosome mobility is expected to be high and hence, comparison of the three biological replicas should provide an “upper limit” of “noise” in nucleosome positioning at this stage. Using this criterion we grouped genes into two classes: i) “noisy” (the genes that change their nucleosome coverage profile around TSSs in at least one replica) and ii) “noiseless” (genes without any changes in the coverage profiles around TSSs among replicas).

Nucleosome profiles are conserved in more than 87% of TSSs and 60% of ORIs among replicas. Yet, only 47% of TSSs and 24% of ORIs can be classified into the same nucleosome architecture family (i.e. sharing the same −1/ + 1 nucleosome positioning and NFR annotation; see *Methods* and Sup. Dataset 1 for details). Notably, NFR width is mostly conserved among replicas at both TSSs and ORIs, confirming that NFR is the most stable property of nucleosome architectures^17^. Finally, the noise level in nucleosome architectures is higher around ORIs than around TSSs (41.5% of ORIs show significant replica-dependent changes compared to 23.9% of TSSs) (Fig. 1e), confirming loose chromatin-dependent regulation around ORIs.

### Functional implications of basal nucleosome architecture

We could not detect any significant difference in gene expression at G1 phase between “noisy” and “noiseless” genes, but genes with expression fold-change greater than 1.2× between G1 replicas are in general “noisy” in terms of nucleosome architecture around TSSs (z-test p-value = 0). Moreover, genes with *M—W* nucleosomal architecture (i.e. with −1 missing and +1 well-positioned nucleosomes) around TSSs typically show high expression level (Welch two-samples test p-value < 1 × 10^−10^). In line with this observation, highly expressed genes tend to have wider NFRs (in average 17 bp longer, p value < 1 × 10^−12^) (Fig. 1g), less phased upstream nucleosomes (p-value 7 × 10^−3^), in agreement with biological intuition^8^,^50^, and also, better-positioned downstream nucleosomes (p-value = 10^−4^) (Fig. 1f). Furthermore, we found H2A.Z enrichment (a known marker of transcription activation) at +1 position of the classes with high expression level : “open” (wide NFR) and *M—W* families, while such enrichment was not observed in “closed” (narrow NFR) families (t.test p-value < 2.2 × 10^−16^; Sup. Figure S3). Altogether, our data clearly demonstrate that loose nucleosome structure in promoters favors activation of gene expression^8^,^50^, and well-positioned nucleosomes in the coding regions are fingerprints of highly expressed genes, indicating that actively transcribed regions require highly ordered chromatin structure for faithful transcription.

Our highly accurate nucleosome maps show that TATA-containing and TATA-less genes (TATA+ and TATA-, using Basehoar classification^51^) show different basal nucleosome architectures. TATA+ genes have fuzzier −1 (p < 10^−10^) and +1 nucleosomes (p < 10^−14^) (Sup. Figure S4a), while TATA- genes are highly enriched in open NFRs (p < 10^−10^) (Sup. Figure S4b). These findings agree well with previous suggestions on a different regulatory mechanism for TATA+ and TATA− genes^52^, and propose that nucleosome architecture plays a major regulatory role for TATA- genes, while other specific protein-DNA contacts may be dominant in the regulation of TATA+genes.

### Cell-cycle dependent nucleosome plasticity at TSSs

**Figure 2:**
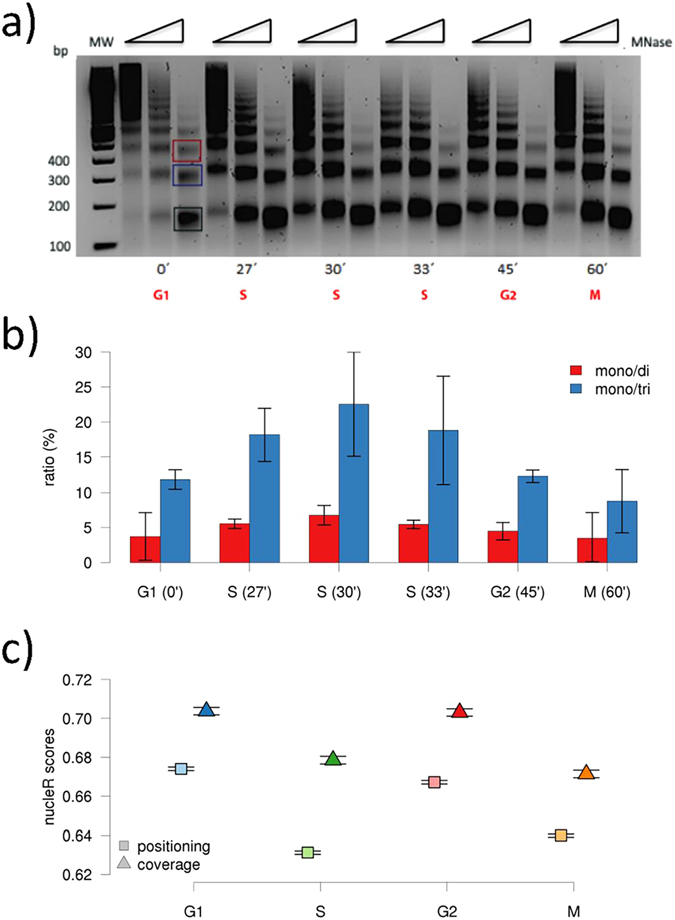
Chromatin sensitivity to MNase digestion along cell cycle. (**a**) Chromatin collected at indicated time points after alpha factor release was formaldehyde cross-linked and digested with increasing amounts of MNase (0.005, 0.01 and 0.025 U) for 25′ at 37 °C, as indicated by the triangles above the lanes. De-crosslinked nucleosomal DNA was separated on 2% agarose gel to compare the digestion pattern along cell cycle. (**b**) Ratio of mono- to di- and mono- to tri-nucleosomes, calculated using IMAGEJ, of MNase digested cell cycle samples from two biological replicas. Error bars signal the 95% confidence interval. (**c**) The mean positioning and coverage scores, based on nucleR score, of −1 and + 1 nucleosomes are shown along cell cycle. Error bars (only visible by the end of the horizontal crossbar) signal the 95% confidence interval.

In order to study the global variations in chromatin organization throughout cell cycle, we have compared MNase digestion patterns and nucleosome profiles of synchronized cell populations at G1, S, G2 and M phases. As shown in Fig. 2, DNA accessibility, determined by the ratio of mono-/tri-nucleosome signals in MNase-digested chromatin, is higher at S phase compared to G1 and G2 gap phases (where RNA and protein synthesis levels are high). On the other hand, chromatin at M phase displays the lowest mono/tri-nucleosome ratio, indicating higher compaction, in agreement with mitotic chromatin condensation. Moreover, nucleosomes are better localized at gap phases than at S and M phases along the genome (see Fig. 2c). These results highlight that cell cycle indeed alters global nucleosome architecture in a way that nucleosome localization is highly affected by DNA replication and, quite surprisingly, by chromatin compaction at mitosis.

**Figure 3:**
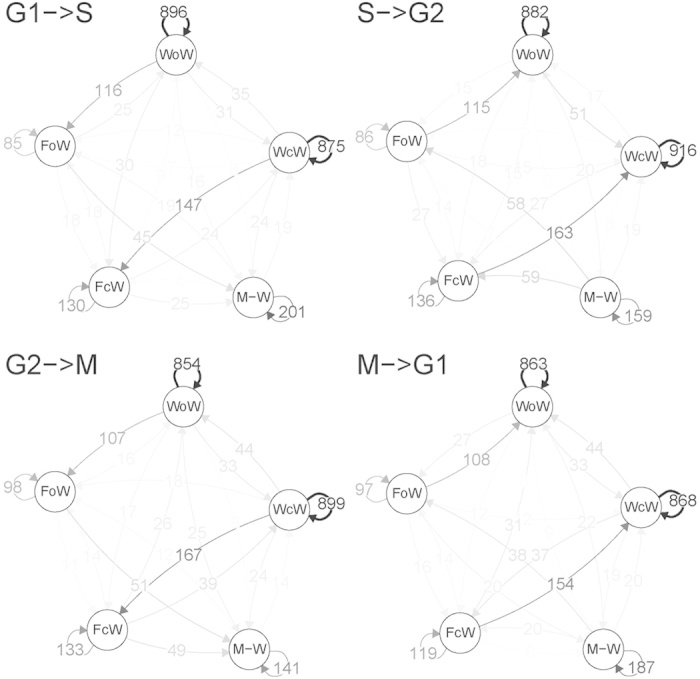
The transition of the nucleosome clusters of the plastic genes along cell cycle. Directional graphs show the number of genes that keep or change the nucleosome architecture around the TSSs (see Methods for clustering details and description) at four transitions along the cell cycle (G1-S, S-G2, G2-M, M-G1).

As next, we have further categorized the analyzed genes according to their nucleosome architecture variations at TSSs along the cell cycle. We have classified the set of genes with conserved patterns as “stable”, those with changes in family annotation as “plastic”, and those with substantial profile changes (corr + ICDB) as “mobile” (around 10% of the genes analyzed) (Sup. Dataset 1; see *Methods*). Accordingly, 1617 genes (representing 49% of total analyzed genes) are “plastic”, albeit most of changes detected along the cell cycle in these “plastic” genes are rather small. About 63% of “plastic” genes show nucleosome variations only in one of the cell cycle stage (i.e. nucleosome architecture is maintained in 3 of the 4 stages). These nucleosome changes mostly take place at S or M phases, where there is an increase in nucleosome fuzziness (Fig. 2c). Furthermore, global variations observed in the G1→S transition are mostly reverted in the S→G2 transition, and similarly, those taking place in G2→M transition are reverted in the M→G1 transition (Fig. 3). Hence, this perfectly orchestrated “plasticity” detected here cannot be simply due to lack of accuracy in nucleosome profiles, which would otherwise lead to random variations. Quite interestingly, “plastic” and “mobile” genes are often “noisy” (75% of “plastic” genes are also “noisy”, compared to 41% of “mobile” and 13.38% of stable genes), indicating that cell cycle-coupled variations are related to intrinsic nucleosome dynamics and suggesting that plasticity and noise are often coupled.

The chromatin remodelers are generally considered to have a large impact on nucleosome rearrangements^53^,^54^. However, our genome-wide analysis reveals that genes with variable architectures are mostly absent of chromatin remodelers (p value = 0 for a two population z test comparison). In fact, nucleosome profiles around TSSs are much better defined in genes bound to chromatin remodelers (Sup. Figure S5). Our data supports the suggestion by Struhl and Segal that ATP-dependent chromatin remodelers might contribute to define the NFRs and hence facilitate nucleosome phasing^24^. Our results suggest that chromatin remodelers would act as restraints to maintain well-positioned nucleosomes and hence, keep chromatin in a “loaded-spring” situation, which can then easily undergo alternative arrangements in the absence of remodeler proteins.

### Cell-cycle dependent nucleosome plasticity at ORIs

Our nucleosome profile comparison around ORIs along the cell cycle reveals that 62 of the 253 annotated replication origins show stable nucleosome organization, while 150 are “plastic”, and 101 are “mobile” (see *Methods*). In contrast to TSSs, where nucleosome changes are mostly periodic between gap and S / M phases, ORI nucleosome architecture shows a more intricate choreography. The widest ORI-NFRs are attained in G1, when pre-replication complex (pre-RC) is formed, while the narrower NFRs are found in S and G2 phases, when the pre-RC dissociate from the chromatin (Sup. Figure S6), supporting the notion that pre-RC activation causes important alterations on nucleosome architecture at ORIs. Moreover, early firing ORIs display better-positioned nucleosomes around ORIs throughout cell cycle than late firing origins (Fig. 1h), indicating regulatory role of nucleosomes on replication activity of early origins. Similar to our observations around TSSs, a significant part of plastic ORIs are detected also as noisy in the G1 phase replicas (116 out of 150 “plastic” and “noisy” χ^2^ p-value < 0.002), highlighting once more that the genome adopts intrinsically fuzzy architectures to modulate biologically relevant changes.

### Functional implications of cell-cycle dependent nucleosome plasticity

**Figure 4:**
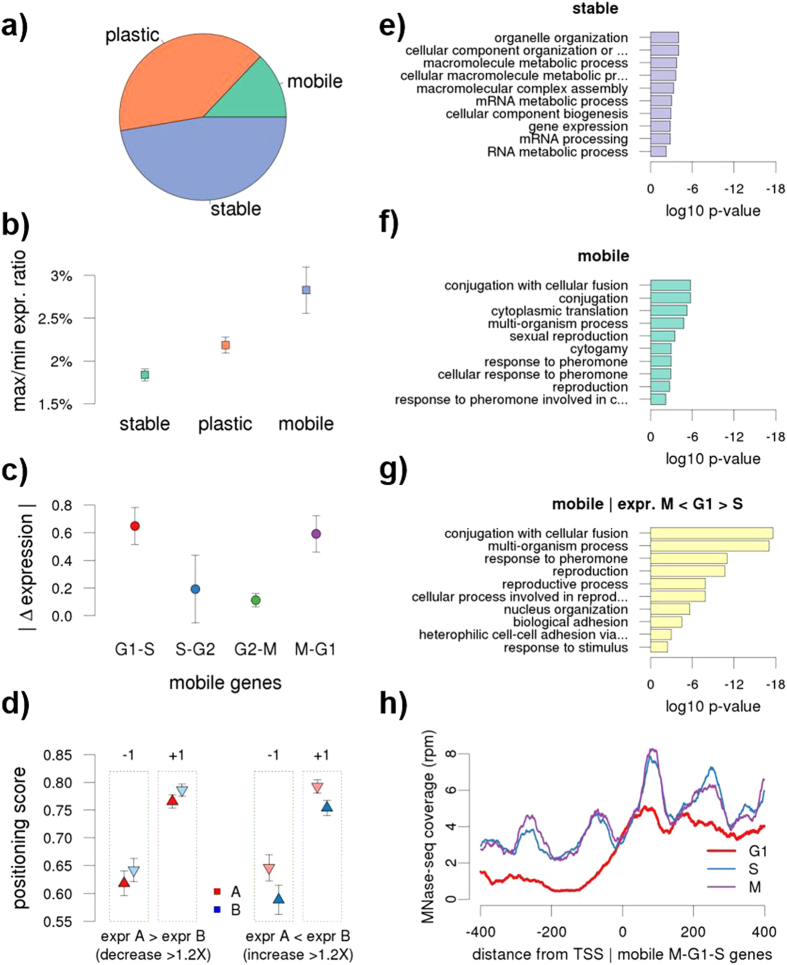
Functional implications of chromatin dynamics along cell cycle. (**a)** Distribution of stable, plastic and mobile genes. **(b**) Ratio between maximum and minimum mRNA expression levels observed during cell cycle for stable, plastic and mobile genes. Error bars indicate 95% CI. (**c**) Absolute expression difference between mobile genes in adjacent stages. Error bars indicate 95% CI. (**d**) Differences in positioning scores of −1/ + 1 nucleosomes relative to the TSSs for genes that decrease (left) and increase (right) their expression in adjacent stages (ie, if A = G1 stage then B = S stage, if A = G2 then B = M, stage). Only genes with an expression fold change of at least 1.2x between considered stages were selected. GO enrichment for (**e**) stable, (**f**) mobile genes. Shown elements represent top 10 terms according enrichment p-value (FDR corrected, minimum significance allowed 0.01). Similar terms have been reduced using ReviGO^63^ (see Methods). (**g**) Average nucleosome coverage around TSSs of mobile genes with higher expression in G1 compared to M and S stages. (**h**) GO enrichment of mobile genes with higher expression in G1 compared to M and S stages

In general, genes with “stable” nucleosome architectures around TSSs are significantly less prone to change their expression along the cell cycle based on the ratio of max to min expression values of each set of genes (Fig. 4a,b). GO analysis shows that stable genes are enriched in cell homeostatic functions such as mRNA processing and organelle organization (odds ratio (OR) from 1.28 to infinite; Benjamini & Hochberg (BH) adjusted p-values between 10^−3^ and 10^−6^; Fig. 4e and Dataset 2). On the contrary, mobile genes have higher variability in the gene expression along the cell cycle and are largely enriched in cell division processes such as conjugation (OR around 10, BH p value < 10^−8^), sexual reproduction and pheromone response (OR between 3–8, BH p-value between 10^−3^ and 10^−5^) (Fig. 4f and Dataset 2). A focused analysis on individual cell cycle transitions reveals that mobile genes in G1→S or M→G1 transitions typically correlate with higher expression changes at these transitions, demonstrating that nucleosome fluctuations at the entry into (M→G1) or the exit from (G1→S) G1 stage have the strongest impact on gene expression (Fig. 4c). In general, the increase in expression in one phase is associated to a decrease in nucleosome coverage and positioning at the corresponding phase compared to the adjacent one (Fig. 4d). Moreover, the specific analysis of MNase-seq coverage revealed that mobile genes with the peak expression at G1 phase have lower coverage and phasing at G1, comparing to adjacent S and M phases (Fig. 4h). GO analysis of this set of genes with higher expression at G1 phase shows that they are enriched in cell division functions (Fig. 4g and Dataset 2).

As noted above, “plastic” or “mobile” genes are often “noisy” at G1 phase, i.e. nucleosome re-arrangements (at TSSs) along the cell cycle take place typically in genes that are intrinsically dynamic at G1 phase. However, the nucleosome architectures of a small subset of “mobile” genes (24) are noiseless at G1 phase. These genes mostly show alterations in nucleosome architecture at the entry into (G2→M) or the exit from (M→G1) mitosis, and are enriched in a variety of functions related to the cell cycle (OR often above 100; associate BH p-values around 10^−4^) (Dataset 3). 15 out of these 24 mobile + /noisy- gene promoters are known to bind chromatin remodeling proteins^55^, suggesting that their nucleosome organization might be tightly controlled by the remodelers during cell cycle to allow large arrangements.

**Figure 5:**
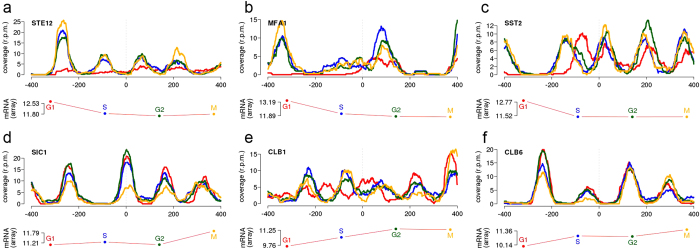
Nucleosome coverage at TSSs and expression levels along CC for genes (**a**) STE12, (**b**) MFA1, (**c**) SST2, (**d**) SIC1, (**e**) CLB1 and (**f**) CLB6. In each panel, a MNase-seq coverage plot is shown for the G1 (red), S (blue), G2 (green) and M (yellow) phases. Gene expression levels are indicated as log2 values of the hybridization ratios from the Affymetrix GeneChip Yeast Genome 2.0 arrays. Expression levels for the four stages of the corresponding genes are shown with the same color code. Note that horizontal axis in mRNA plot is not aligned anyhow with the upper plot. Genes in the first row (**a–c**) are related to alpha-factor response and sensibilitzation. Bottom three (**d–f**) are genes involved in other cell cycle functions.

Overall, our analyses suggest a strong connection between gene expression and nucleosome architecture for a reduced, but functionally important, set of genes. This link is, as expected, particularly evident for the genes involved in the alpha-factor mating pheromone response pathway (see Sup. Figure S7). Similar plots for all genes are available at http://mmb.pcb.ub.es/cell_cycle2014). Alpha-factor activates Ste2 expression, which is coupled with a total eviction of nucleosomes in the upstream TSS region and a significant increase in + 1 nucleosome fuzziness. Ste2, in turn, activates heterotrimeric G-protein and eventually upstream MAPK cascade components, including Fus3, which stimulates the synthesis of Ste12 and Far1 to promote cell cycle arrest at G1 phase. As shown in Fig. 5 and Sup. Figure S7, the activation of these four genes is coupled with an almost complete eviction of −1 and + 1 nucleosomes. In addition, genes encoding the alpha-factor (MFA2 and MFA1) or regulating desensitization to alpha-factor (such as SST2) are highly transcribed in G1 arrest and exhibit a dramatic nucleosomal reorganization of the promoter region in this stage (Fig. 5 and Sup. Figure S7).

Cell cycle-regulatory genes are the second major group showing nucleosome activity coupled to gene expression. For instance, Sic1, which prevents premature S-phase entry, shows maximum expression at M phase, where the −1, + 1 and even +2 nucleosomes have lower occupancy (Fig. 5). Inhibition of Sic1 allows Clb6 activation, which is coupled with a large change in the −1 and −2 nucleosomes at M phase (Fig. 5). Clb6 participates in DNA replication initiation at M/G1 phase by activating helicase maintenance (MCM) proteins to unwind DNA. The activation of MCM3 and MCM7 subunits is coupled to an enlargement of the NFR. Subsequently, MCM1 regulates several genes that exhibit a similar temporal expression pattern from late S to M phase: SWI5, ACE2, and CDC5^56^. Not surprisingly, their nucleosome profiles rearrange in the inactive G1 phase, either by a change of phase in the −1/ + 1 nucleosomes or by a higher occupancy/positioning of the upstream −2 nucleosome (Sup. Figure S8).

### Discussion and conclusions

The alterations in chromatin organization has been demonstrated to be related to cell cycle events, emphasizing their role on the cell cycle regulation^57^. However, it is still unclear whether these variations are conducive to specific cell cycle activities. Therefore, through our comprehensive analyses we studied the nucleosome plasticity along the yeast cell cycle to explore its functional role on gene expression and DNA replication.

In general, cell cycle-dependent variations in nucleosome pattern around TSSs take place in a cyclic manner with good coordination between G1/G2 and S/M phases. Chromatin at gap phases show better localized nucleosome architectures, while S and M phase-chromatin display fuzzier nucleosomes. On the other hand, NFR width is quite conserved along the cell cycle, suggesting that NFRs are not affected by cellular processes taking place along the cell cycle. The static status of NFRs highlights the underlying role of DNA physical properties on its determination.

The cell cycle-dependent fluctuations of nucleosome pattern around ORIs are more complex than around TSSs. The highest variations are observed at G1 phase when pre-RC is formed. Recruitment of replicatory proteins at this phase probably leads to preclusion of nucleosome accessibility to longer DNA segments, resulting in wider NFR at G1 phase. On the other hand, nucleosome positioning is more robust in G2, when pre-RC is already disassembled, supporting the notion that pre-RC activation leads to important nucleosome rearrangements at replication origins. In addition, early firing origins show better-positioned nucleosomes and more stable architecture along the cell cycle than the late origins, suggesting the tighter regulatory role of nucleosome on activity of early replication origins for faithful DNA replication.

Our analysis further revealed new insights into the interplay between transcription and nucleosome organization. We demonstrated that the genes with depleted nucleosomes upstream of TSSs or with wider NFRs have in general high transcription level. These highly expressed genes show mainly increased nucleosome density in the coding region, demonstrating that well-organized nucleosome assembly and disassembly is required for the actively transcribed regions perhaps to aid in RNA polymerase movement.

Genes with “stable” nucleosome architecture along cell cycle show also static expression level along the cell cycle and are enriched in cell homeostatic functions. On the other hand, “mobile” genes tend to change their expression level along the cell cycle, and they are enriched in cell-cycle related functions, demonstrating the interplay between transcription and nucleosome positioning. The detailed analysis of a small number of well-characterized genes shows the coordination between nucleosome choreography and gene activity, confirming the tight connection between chromatin architecture and cell-cycle dependent functionality.

The link between noise and cell-cycle dependent mobility in nucleosome architecture strongly suggests that cells typically use the intrinsic dynamic nature of nucleosomes to mediate chromatin changes required for cell cycle progression. On the other hand, the role of chromatin remodelers seems to be mostly to maintain highly ordered nucleosome structures rather than to define mobile architectures, suggesting that they might keep the chromatin in a “spring-loaded” conformation.

Overall, our detailed analyses provide a comprehensive description of nucleosome plasticity along cell cycle and also show how this determines the expression changes and replication activity, highlighting the role of nucleosome architecture in genome function.

## Methods

### Cell-cycle synchronization

Yeast strain BY4741 was grown using fresh YPD media at 30 °C until an OD_600_ of 0.2. Then, alpha-factor mating pheromone (GenScript) was added to the culture to a final concentration of 10 μM and the culture was incubated for 2 h to induce cell-cycle arrest in late G1. The alpha-factor was removed by harvesting the cells with centrifugation for 10′ at 4500 g. The samples were collected every 10´after alpha factor release.

Cell synchrony was monitored by three approaches: flow cytometry (FACS), fluorescence microscopy and budding index calculation (see Sup. Figure S1). For FACS analysis, cells were fixed with 100% EtOH, spun down and washed once with 1 × SSC buffer (150 mM NaCl, 15 mM sodium citrate, pH 7.80). Removal of RNA and proteins were carried out by incubation with RNase A (0, 5 mg/ml, Roche) and Proteinase K (0.5 mg/ml, Roche), respectively. Samples were briefly sonicated by using the Bioruptor system and mixed with 500 μl SSC buffer containing 0.1 mg/ml propidium iodide (PI, Sigma-Aldrich). Fluorescence emitted from DNA-intercalated PI was measured by Beckman Coulter EPICS^®^ XL flow cytometer. Cell-cycle progression was also monitored by fluorescence microscopy and budding index calculation. For these purposes, cells were briefly sonicated and fixed with EtOH in a similar manner as for FACS. Fixed cells were then resuspended in 200 μl PBS containing Hoechst stain at 30 μg/ml. Finally, cells were placed on a glass slide and visualized by fluorescence microscopy (Nikon E600 microscope). For budding index calculation, a sample from EtOH-fixed cells was placed on a hemocytometer and visualized under a phase contrast microscope to count the number of budded and unbudded cells (see Suppl. Figure S1).

### Nucleosomal DNA extraction

The collected samples that correspond to G1, S, G2 and M phases (0′, 30′, 45′, 60′) were fixed immediately with 700 μl of 37% formaldehyde and nucleosomal DNA was prepared as previously described^14^. The overall nucleosome digestion was accurately controlled by carrying out several digestion reactions with MNase at concentrations of 0.04, 0.08, 0.12 and 0.16 U, at 37 °C for 30 min to optimize chromatin fragmentation. Reactions were stopped by addition of EDTA to a final concentration of 0.02 M and subsequently incubated with RNase A (0.1 mg) for 1 h at 37 °C and further treated with Proteinase K at 37 °C for 1 h. DNA was extracted using phenol–chloroform extraction and concentrated by ethanol precipitation. The percentage of mononucleosomal DNA fragments was examined by means of 2% agarose gels. The integrity and size distribution of digested fragments were determined using the microfluidics-based platform Bioanalyzer (Agilent) prior to DNA sequencing. Typically, samples containing >80% mononucleosomal fragments, digested with 0,08 U, were sent for sequencing.

Cleaved DNA samples were sequenced with Illumina *HiSeq 2000*, obtaining between 50 M and 90 M paired reads per experiment. Sequencing experiments were done in triplicates to minimize potential artifacts and biases. Raw reads are available at the *ENA-SRA* website (http://www.ebi.ac.uk/ena) with accession number PRJEB6970.

### RNA Isolation and gene-expression arrays

Cells were collected at the same intervals as nucleosomal DNA samples in icy-water and harvested by spinning for 3–4 min at 6000 rpm, frozen in liquid nitrogen and stored at −80 °C. Total cellular RNA was extracted using the RNeasy kit (Qiagen), following the manufacturer’s instructions with the spheroplasting protocol (0.5 mg/ml zymolase for 30′). The total RNA was hybridized to Affymetrix GeneChip Yeast Genome 2.0 arrays for gene-expression analysis.

Transcript abundance was measured as the median log_2_ ratio of data from all probes fully contained within the length of the transcript, which identified 4,815 coding transcripts. We defined the fold-change expression for a particular gene in two different cycle phases as the power of unsigned difference between their log2 expression ratios. Raw files available in ArrayExpress under accession number E-MTAB-2839 and E-MEXP-3702.

### Nucleosome mapping and calling

MNase-seq short reads were aligned to the SacCer3 genome (UCSC, downloaded from http://hgdownload-test.cse.ucsc.edu/goldenPath/sacCer3/ bigZips) using Bowtie aligner^58^ allowing up to two mismatches per read. Paired-end reads were matched and coverage was calculated using R/Bioconductor^59^ and nucleR library^60^, yielding to a fold-coverage between 35x−80x. 93.5% of the genome was covered by at least one read and nucleosome calls covered 79.7% of the yeast genome, with an average of 67.000 calls. In order to improve the signal-to-noise ratio, reads were trimmed to their central 50 bases easing the identification of individual peaks in the plots. In order to assess the fuzziness of different peaks, we used the calling score reported by nucleR. Lower scores (score_w < 0.4 & score_h < 0.6) stand for poor positioned or covered nucleosomes (fuzzier calls), while scores closer to 1 are usually well-positioned nucleosomes with a good coverage, except if they overlap with another nucleosome call, in which case they are marked as fuzzy.

### Nucleosome architecture clustering

In order to study the nucleosome architecture in key loci, we used a clustering approach based on the positioning of neighboring nucleosomes, as reported previously^17^. The nucleosome call upstream the reference locus was annotated as −1 nucleosome and the one immediately downstream +1 nucleosome. nucleR scores were used to determine the fuzziness state — well-positioned (W), fuzzy (F) or missing (M). The distance between two reference nucleosomes was defined as NFR and classified as open (o) if the distance between nucleosome dyads was around 270 bp and as closed (c) if it was around 170 bp, following a bimodal distribution (Fig. 1). The combination of < −1 positioning state > − < NFR width > − < + 1 positioning state > defines the clustering group.

Only non-ambiguous classifications are reported in this work (this excludes non-covered areas or overlapping nucleosome calls). Additionally, we considered only the genes that are also covered by the expression array, yielding to a set of 3279 genes with information about nucleosome structure and gene expression.

### ORI annotation

We used the annotation of experimentally validated ORIs from Eaton *et al.*^16^ Position of reported ORIs and ACS were updated to SacCer3 genome using liftOver tool from UCSC (https://genome.ucsc.edu/cgi-bin/hgLiftOver). Replication times for different ORIs were obtained by crossing this dataset with the one provided by Yabuki *et al.*^61^.

### Measure of changes in nucleosome architecture

In order to assess the variations in nucleosome organization between two samples we used two different measures. We considered a qualitative change when the clustering group of a given locus for two samples varies, which also gives information about the directionality of the change. In order to detect large chromatin rearrangements independently from the clustering method, we also defined a numeric measure of the nucleosome dynamics, obtained by combining the correlation coefficient (corr) and the logarithm of the coverage difference per base (lCBD) between two samples. Pearson correlation between windows of −400:400 from the locus of study is able to detect shifts in the coverage profiles, while lCDB detects changes also in the height of coverage peaks. lCDB is defined as:


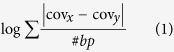


where *cov* is the mean normalized coverage in the previous window for the two reference phases and #bp is the number of base pairs (bp) in such window.

We defined changes based on the clustering classification of different cell cycle stages within the replica as “plastic” architectures and large reorganizations of chromatin based on corr and ICDB (architecture changing, corr <0.7 or lCDB > 0.8) as “mobile”. In a similar manner, variations in the same stage of the cell cycle between different replicas are defined as “noisy”.

### Gene Ontology (GO) analysis

Hypergeometric tests for GO-term enrichment in different gene sets were made using GOstats library^62^. Significance of GO-analysis was assessed by adjusted p-values using Benjamini & Hochberg (BH) corrections for multiple testing. When applicable, data reduction was done using ReviGO web service^63^, selecting those GO terms with relevance score >0.5.

### Calculation of nucleosome deformation energy

The energetic cost of wrapping a 147 bp DNA fragment was determined by using a harmonic approach:


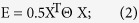


where Θ is the sequence-dependent stiffness matrix derived from atomistic molecular dynamics (MD) simulations^64^,^65^; X (or X^T^) is the deformation vector (or its transposed), given by converting a relaxed DNA fiber into a coiled nucleosome core conformation as described for averaging and smoothing of X-ray structures^66^,^67^,^68^,^69^,^70^,^71^,^72^,^73^,^74^.

## Additional Information

**Accession numbers**: Microarray raw data were deposited in the ArrayExpress (http://www.ebi.ac.uk/arrayexpress/) with accession number E-MTAB-2839 and E-MEXP-3702. Sequencing short reads were deposited in the *European Nucleotide Archive* (http://www.ebi.ac.uk/ena) with accession number ERP001689. All preprocessed coverage data, gene expression values and gene analyses are available in our website http://mmb.pcb.ub.es/cell_cycle2014.

**How to cite this article**: Deniz, O. *et al.* Nucleosome architecture throughout the cell cycle. *Sci. Rep.* **6**, 19729; doi: 10.1038/srep19729 (2016).

## Supplementary Material

Supplementary Information

Supplementary Dataset 1

Supplementary Dataset 2

Supplementary Dataset 3
