# Supplementary material for: Nucleosome architecture throughout the cell cycle
Source: Sci Rep. 2016 Jan 28;6:19729. doi: 10.1038/srep19729 (PMC4730144; doi:10.1038/srep19729)
Supplement: Supplementary Information [file srep19729-s1.pdf]

Nucleosome architecture throughout the cell cycle

Özgen Deniz [1,2,5&], Oscar Flores [1,2,6&], Martí Aldea [3], Montserrat Soler-López [1,2,7] and Modesto Orozco [1,2,4*]

Supplementary Figures & Dataset Captions

---

1 Institute for Research in Biomedicine (IRB Barcelona). Baldiri Reixac 10-12. 08028 Barcelona, Spain
2 Joint BSC-CRG-IRB Program in Computational Biology. Baldiri Reixac 10-12. 08028 Barcelona, Spain
3 Molecular Biology Institute of Barcelona (IBMB) CSIC. Baldiri Reixac 4. 08028 Barcelona, Spain
4 Department of Biochemistry and Molecular Biology. University of Barcelona, 08028 Barcelona, Spain
5 Present address: Blizard Institute, 4 Newark Street E1 2AT, London, UK
6 Present address: Genomcore SL, Jordi Girona 1, 08034 Barcelona, Spain
7 Present address: European Synchrotron Radiation Facility (ESRF), 38000 Grenoble, France

SUP FIGURE S1

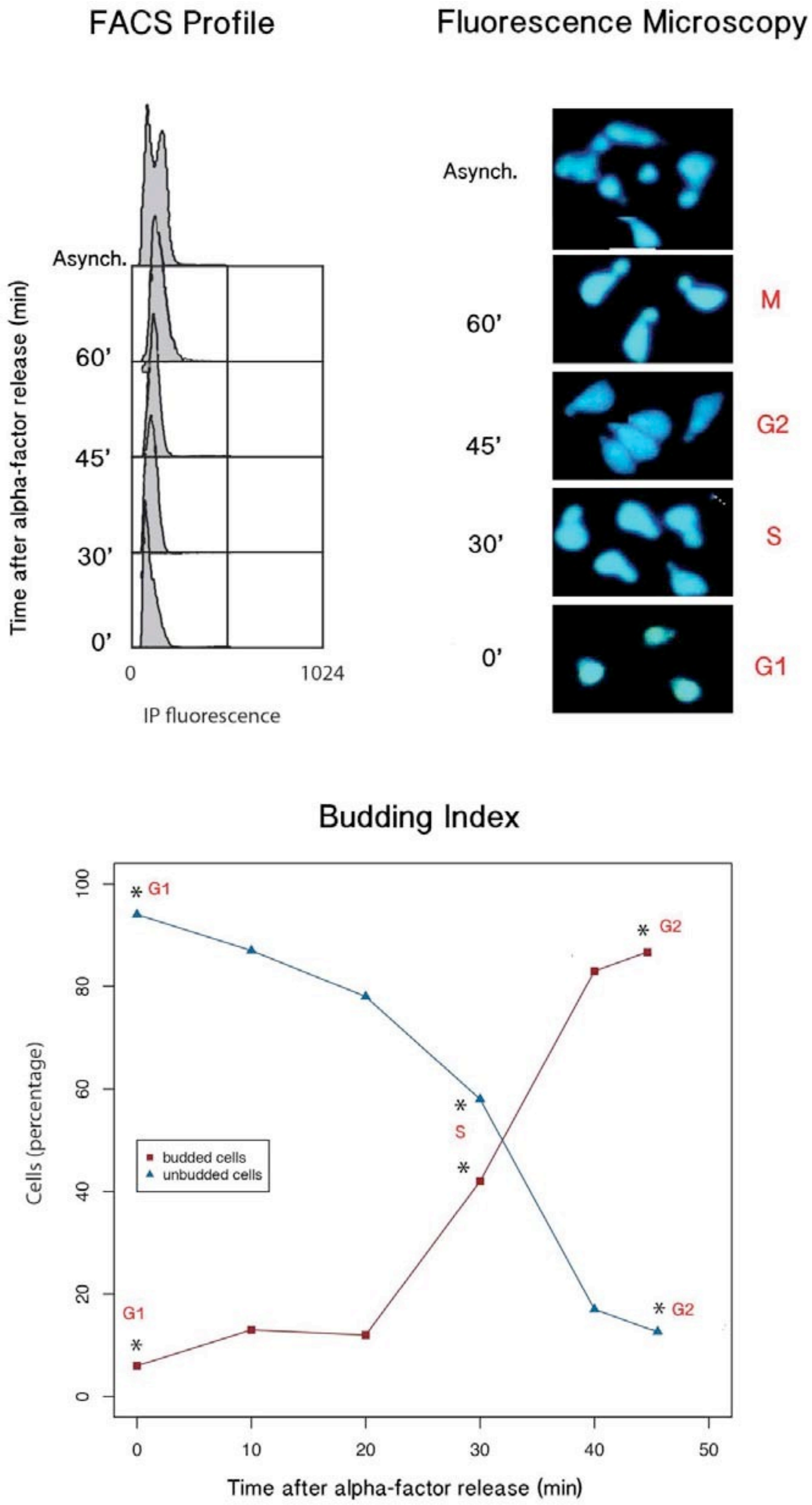

Supplementary Figure S1 – Monitoring cell cycle progress of BY4741 cells, by flow cytometry (top left), fluorescence microscopy (top right) and budding index (bottom).

SUP FIGURE S2

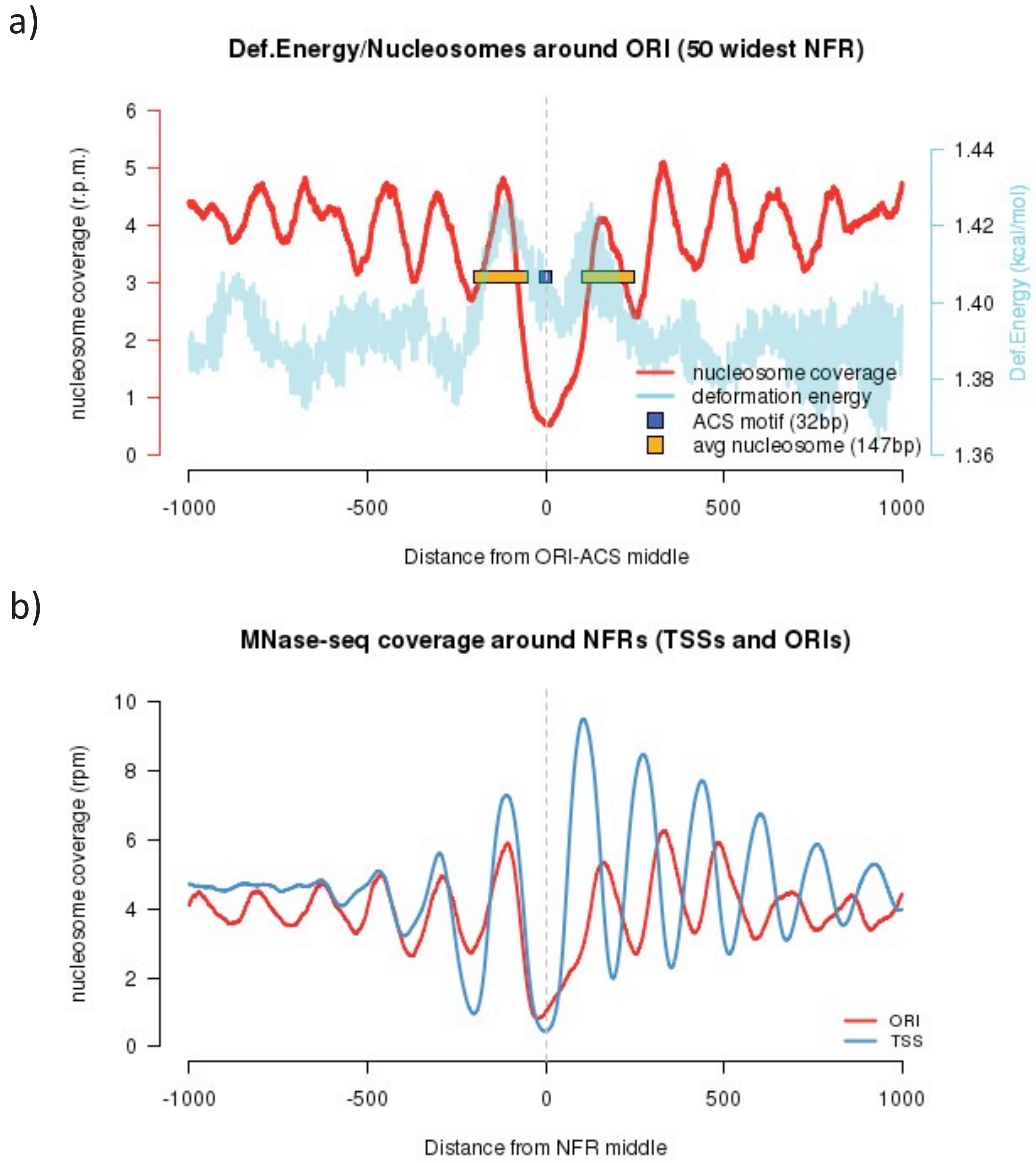

Supplementary Figure S2 – Properties of the NFRs at ORIs. **a)** The deformation energy (blue) and coverage maps (red) were calculated and averaged across the widest NFRs at ORIs (top 50). ORI-NFRs are signaled by specific physical properties of the underlying DNA sequence (higher deformation energy shown in blue), in a similar manner to TSS-NFRs (Flores et al, 2014) **b)** Distribution of average nucleosome coverage profiles at TSSs (blue) and ORIs (red). Average nucleosome positioning at TSSs are directional and asymmetrical, meanwhile symmetry is more prevalent in the ORIs.

SUP FIGURE S3

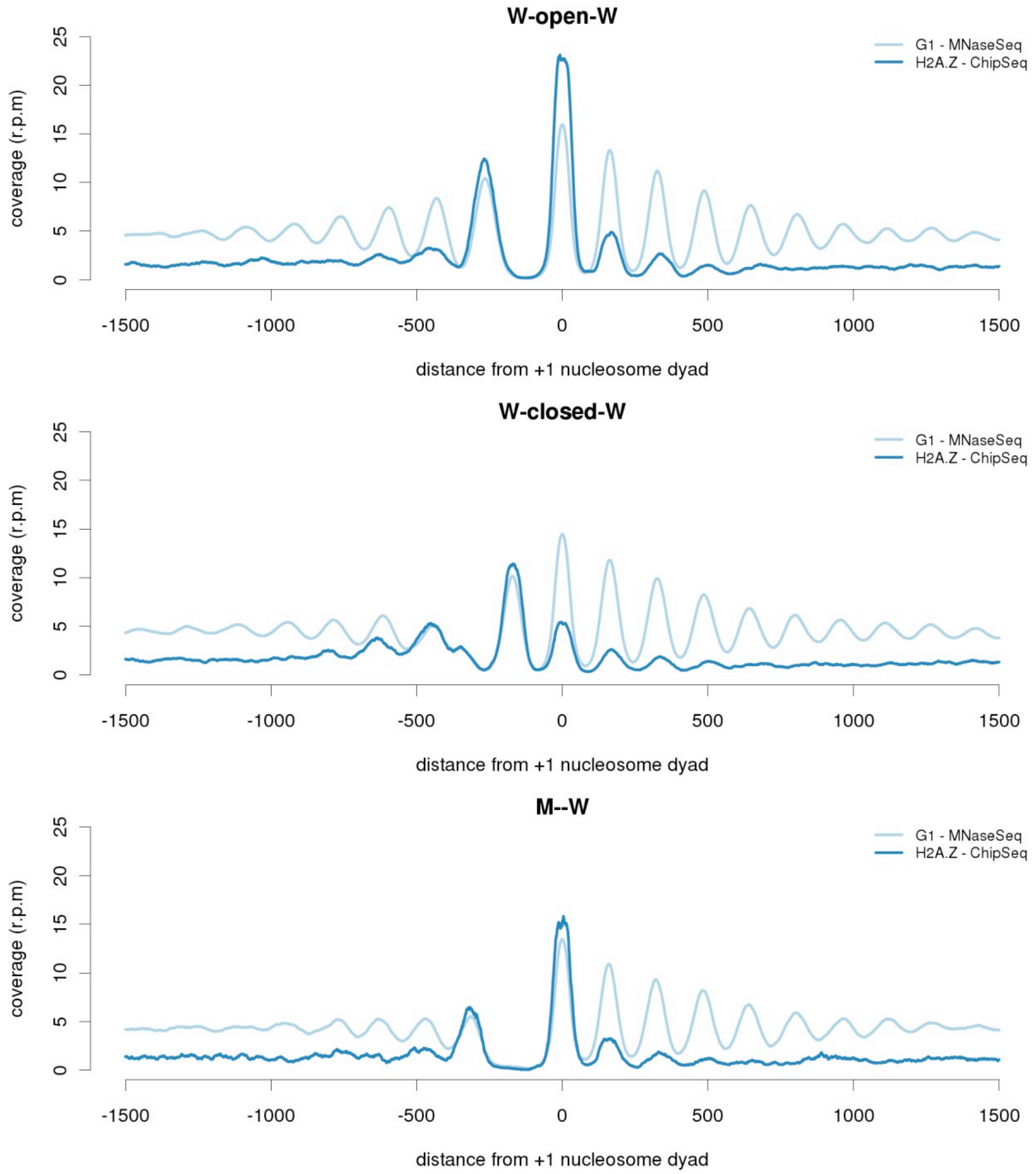

Supplementary Figure S3 – H2A.Z histone variant enrichment in gene clusters. H2A.Z coverage (Albert, 2007) is compared with G1 nucleosome coverage maps in a) open, b) closed and c) missing -1 nucleosome families with flanking well positioned nucleosomes.

SUP FIGURE S4

a)

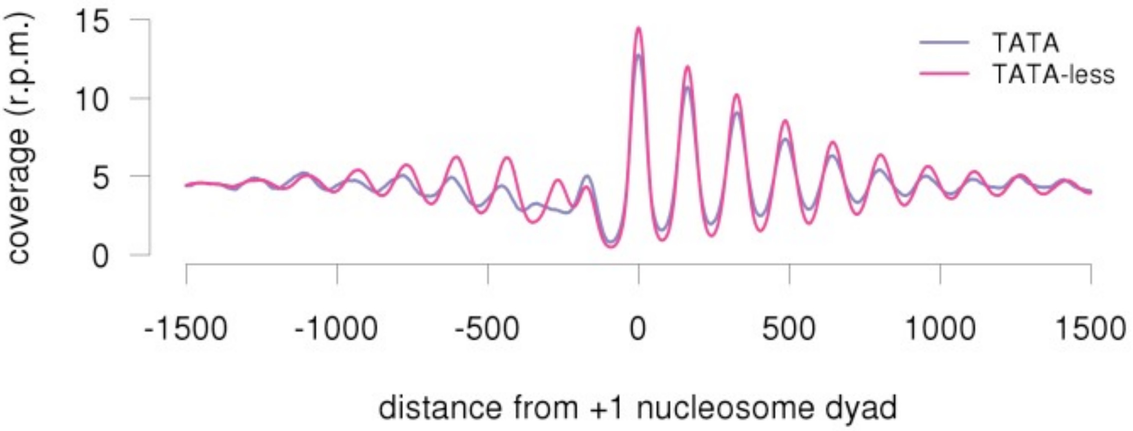

b)

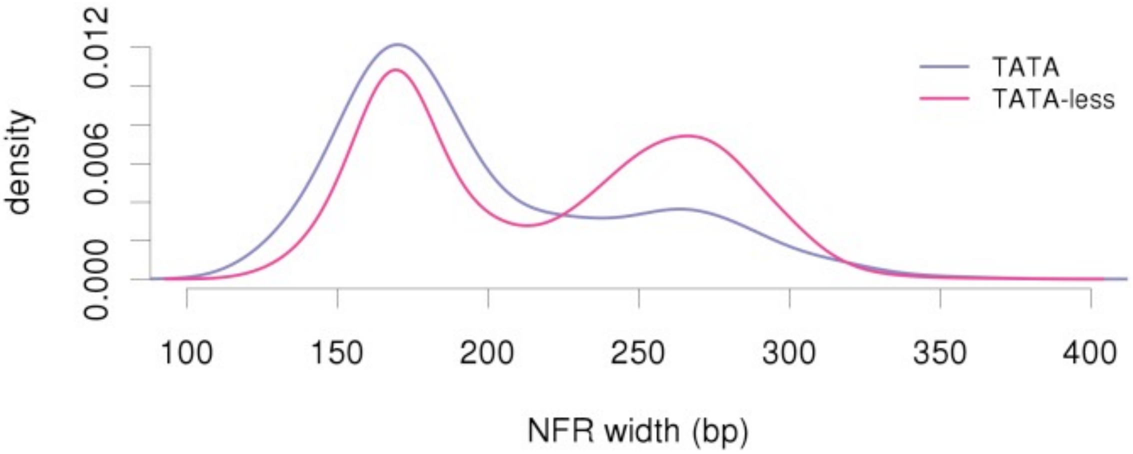

Supplementary Figure S4 – Nucleosome coverage in TATA and TATA-less promoters. Nucleosome coverage maps of TATA-box regulated genes (pink) are compared with TATA-less genes (purple). **a)** Average coverage taking the dyad of the +1 nucleosome as reference does not display any difference in the gene body. **b)** NFR distribution in TATA and TATA-less promoters.

SUP FIGURE S5

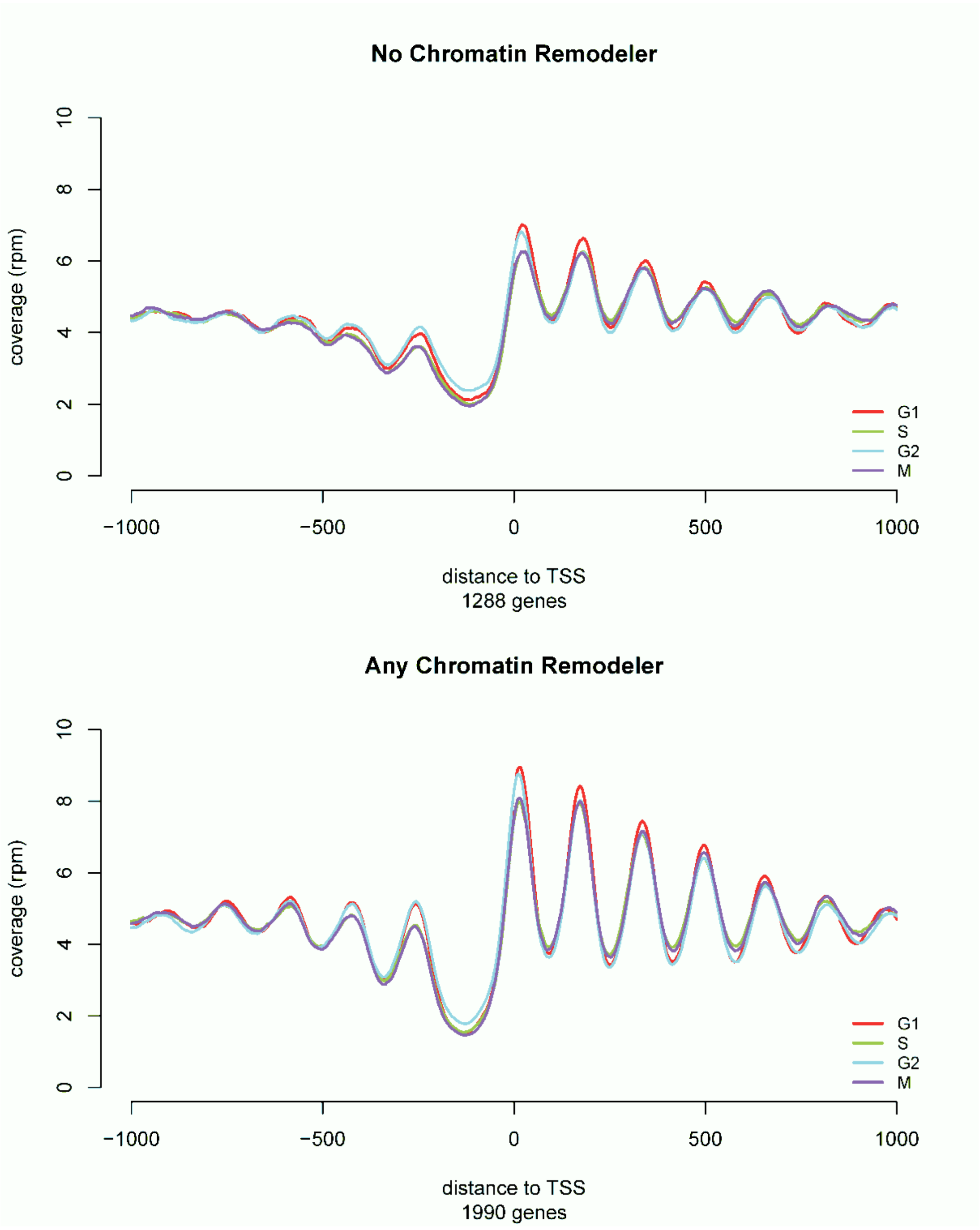

Supplementary Figure S5. Effect of chromatin remodelers in nucleosome architecture. Average nucleosome coverage at TSSs for genes not bound by any chromatin remodelers (top) and bound by any chromatin remodeler (bottom). The genes regulated by remodelers display a better phasing of nucleosome arrays.

SUP FIGURE S6

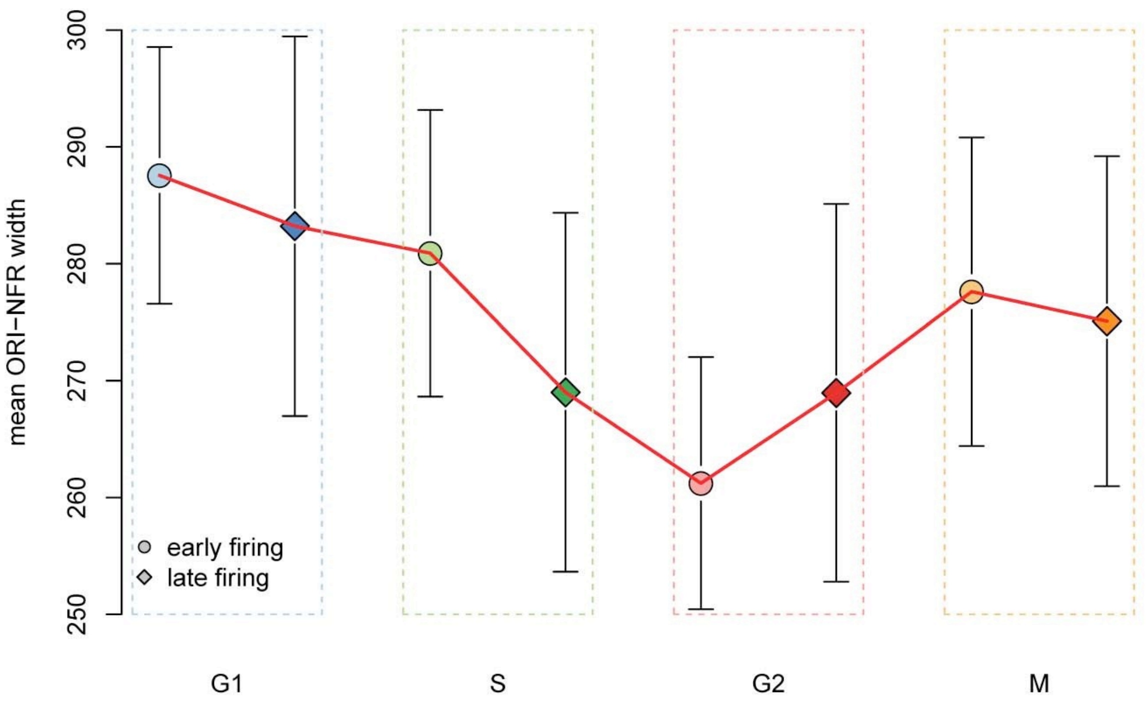

Supplementary Figure S6 – **Nucleosome free region at ORIs along cell cycle**. Average width of ORI-NFRs changes during cell cycle, both in early and late firing ORIs. Error bars signal the 95% confidence interval, which is wide due to the low number of observations (N=100 in both groups)

SUP FIGURE S7

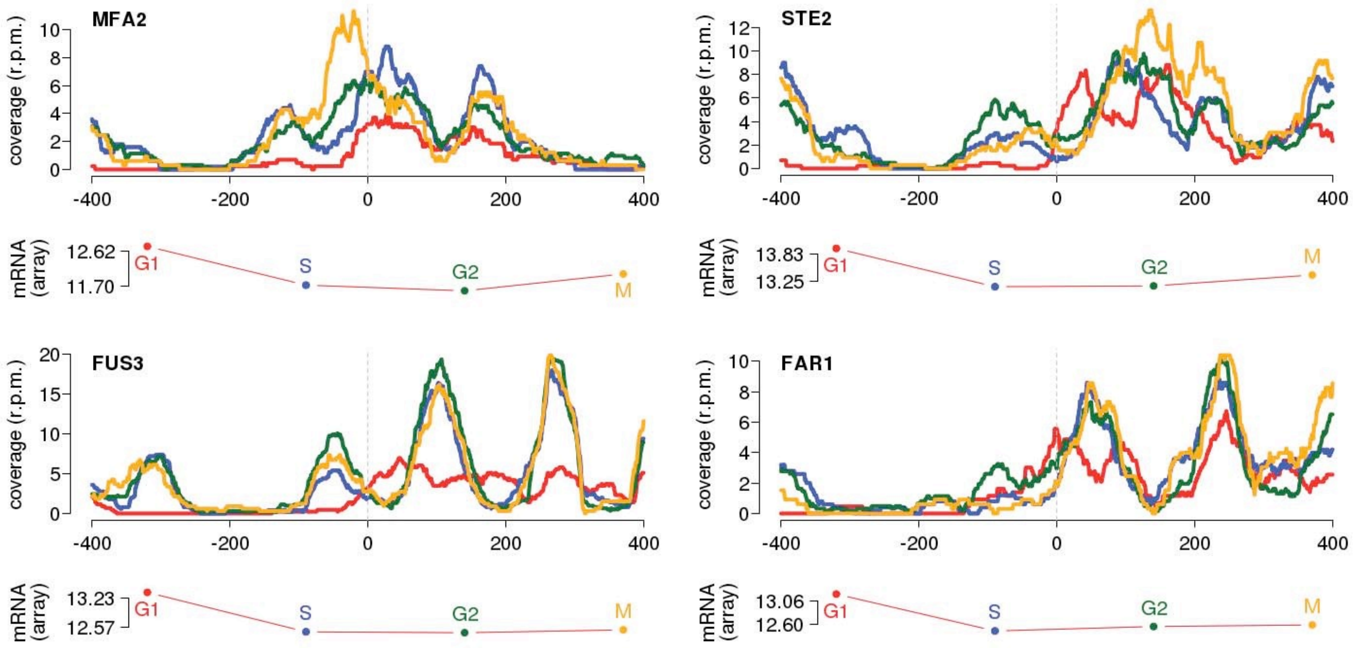

Supplementary Figure S7 – **Nucleosome architecture and expression plasticity of genes involved in alpha-factor pheromone mating and its desensitization response.** Up to bottom and left to right): MFA2, STE2, FUS3 and FAR1. Gene expression levels are indicated as log2 values of the hybridization ratios from the Affymetrix GeneChip Yeast Genome 2.0 arrays.

SUP FIGURE S8

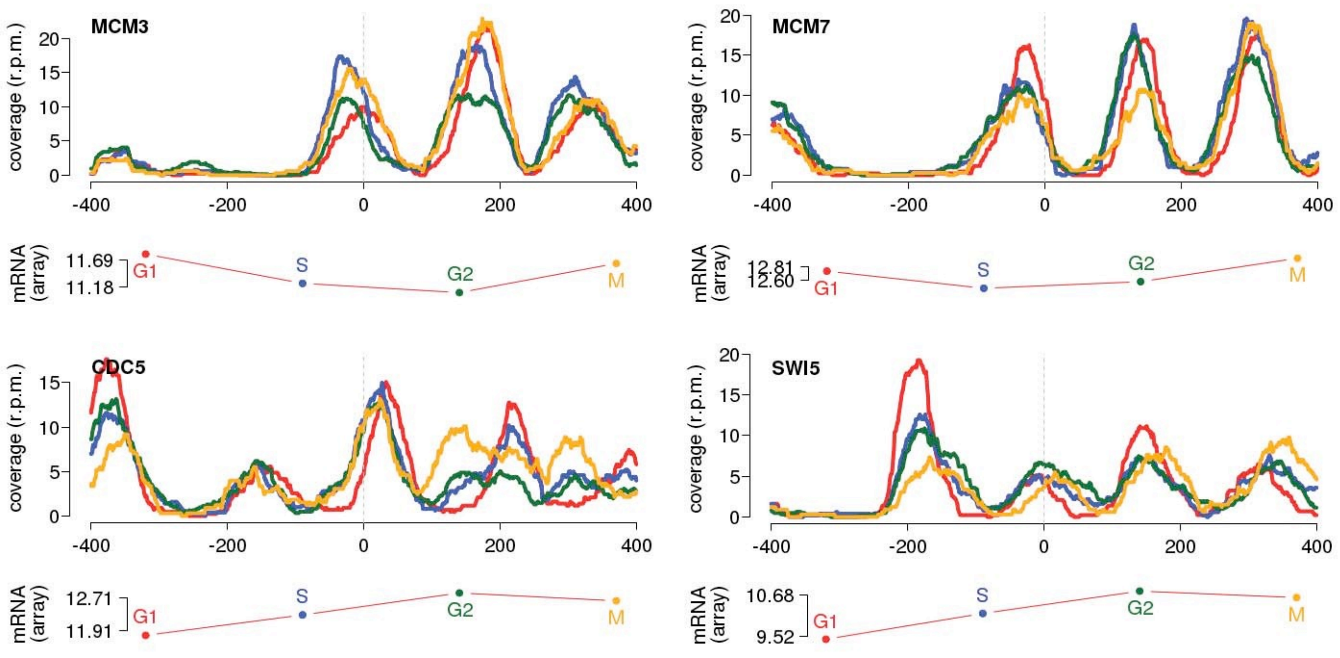

Supplementary Figure S8 – Nucleosome architecture and expression plasticity of genes involved in cell cycle progression. Up to bottom and left to right): MCM3, MCM7, CDC5 and SWI5. Gene expression levels are indicated as log2 values of the hybridization ratios from the Affymetrix GeneChip Yeast Genome 2.0 arrays.

Dataset captions

Dataset 1. Nucleosome architecture dynamics around TSS during cell cycle

Dataset 2. GO-term enrichment analysis for different gene clusters

Dataset 3. GO-term enrichment analysis for combinations of mobile genes with high expression fold-change (>1.2)
